# AnoChem: Prediction of chemical structural abnormalities based on machine learning models

**DOI:** 10.1016/j.csbj.2024.05.017

**Published:** 2024-05-15

**Authors:** Changdai Gu, Woo Dae Jang, Kwang-Seok Oh, Jae Yong Ryu

**Affiliations:** a Artificial Intelligence Laboratory, Oncocross Co., Ltd., Saechang-ro, Mapo-gu, Seoul 04168, Republic of Korea; b Department of Artificial Intelligence, College of Computing, Yonsei University, 50 Yonsei-ro, Seodaemun-gu, Seoul 03722, Republic of Korea; c Data Convergence Drug Research Center, Korea Research Institute of Chemical Technology, 141 Gajeong-ro, Yuseong-gu, Daejeon 34114, Republic of Korea; d Department of Medicinal and Pharmaceutical Chemistry, University of Science and Technology, Daejeon 34129, Republic of Korea; e Department of Biotechnology, Duksung Women’s University, 33 Samyang-Ro 144-Gil, Dobong-gu, Seoul 01369, Republic of Korea

**Keywords:** AnoChem, Drug design, Machine learning, Computational chemistry, Cheminformatics

## Abstract

*De novo* drug design aims to rationally discover novel and potent compounds while reducing experimental costs during the drug development stage. Despite the numerous generative models that have been developed, few successful cases of drug design utilizing generative models have been reported. One of the most common challenges is designing compounds that are not synthesizable or realistic. Therefore, methods capable of accurately assessing the chemical structures proposed by generative models for drug design are needed. In this study, we present AnoChem, a computational framework based on deep learning designed to assess the likelihood of a generated molecule being real. AnoChem achieves an area under the receiver operating characteristic curve score of 0.900 for distinguishing between real and generated molecules. We utilized AnoChem to evaluate and compare the performances of several generative models, using other metrics, namely SAscore and Fréschet ChemNet distance (FCD). AnoChem demonstrates a strong correlation with these metrics, validating its effectiveness as a reliable tool for assessing generative models. The source code for AnoChem is available at https://github.com/CSB-L/AnoChem.

## 1 Introduction

Lead optimization is a crucial process in drug discovery [1], during which chemical structures are designed to increase potency and improve various molecular properties, including absorption, distribution, metabolism, excretion, and toxicity (ADME/Tox). Since many conditions must be considered in the design process of chemical structures, this is one of the most time-consuming and expensive steps in the early stages of drug development.

Computational drug discovery has accelerated the discovery process by predicting molecular properties and designing chemical structures [2]. In particular, computer-based drug design based on deep learning has become important in lead optimization by suggesting novel chemical structures with the desired properties. Specifically, deep generative models, such as variational autoencoders (VAE) [3], [4], [5] and generative adversarial networks (GANs) [6], [7], have been widely used to generate the chemical structures of drugs with the desired properties and/or chemical scaffolds. These deep generative models, including junction tree-variational autoencoder (JTN-VAE), adversarial autoencoder (AAE), and LatentGAN, have been successfully employed to discover hit compounds by sampling and exploring the chemical spaces of drug-like compounds [8]; they have also been used for the generation of chemical structures with the desired molecular properties in the lead optimization process [9], [10], [11]. For example, generative tensorial reinforcement learning (GENTRL) has been used to design DDR1 inhibitors [12].

Although generative models are widely used in drug design, several issues remain. For example, determining the feasibility of the resulting chemical structures has proven challenging. Currently, quantitative estimates of drug-likeness (QED) [13], synthetic accessibility score (SAscore) [14], logP, and molecular weight (MW) are used to evaluate chemical structures and assess the generated chemical structures and molecular properties. Furthermore, several metrics have been suggested for assessing the performance of models, such as the Fréschet ChemNet distance (FCD), to measure the distance between real and generated molecules. The FCD measures the distance between two chemical structural distributions [15], which is similar to the concept of the Fréschet inception distance (FID) and is widely used to evaluate the performance of generative models. Although several studies have introduced metrics to evaluate the generated molecules, a need persists for the improvement of evaluation metrics [16], particularly with respect to determining the probability of the existence of the generated molecules [3].

**Fig. 1:**
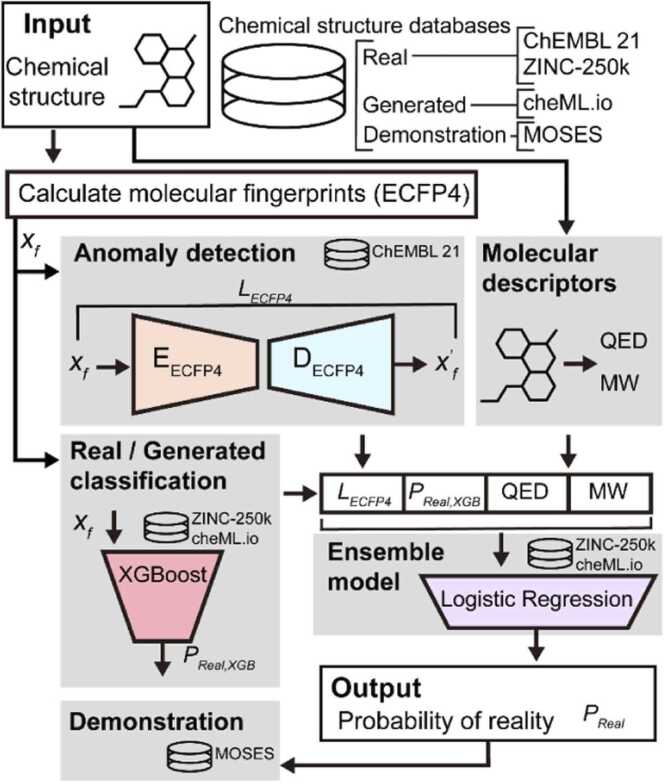
Schematic diagram of AnoChem. AnoChem comprises an anomaly detection model utilizing ECFP4 fingerprint, along with a real/generated classification model based on XGBoost. The logistic regression (LR)-based ensemble model takes the ECFP4 recovery (*L*_*ECFP4*_) from the anomaly detection model, classification result from the classification model (*P*_*Real,XGB*_), and molecular properties of the chemical structures (QED and MW), and the ensemble model generates the final prediction score, *P*_*Real*_, which represents the probability of a generated molecule being a real one. The anomaly detection models are trained on the ChEMBL21 dataset, while the classification and ensemble models are trained on ZINC-250k and cheML.io datasets. AnoChem has been assessed with real and generated chemical structures of the MOSES dataset as an out-of-bag test. Abbreviations: QED, quantitative estimates of drug-likeness; MW, molecular weight; LR, logistic regression; XGBoost, extreme gradient boosting.

To address these issues, we present AnoChem (Fig. 1), a computational framework based on deep learning for predicting anomalies in the chemical structure of an input compound. AnoChem utilizes an anomaly detection model and a binary classification model to distinguish real from generated compounds. Finally, AnoChem predicts the anomaly score of the chemical structure using an ensemble model. This score is then applied to determine whether a compound generated by a model is real. Thus, AnoChem will prove to be a useful tool for filtering invalid molecules generated by deep generative models during the lead optimization process.

## 2 Materials and methods

### 2.1 Datasets

The real chemical structures used in this study were sourced from ChEMBL 21 [17] and ZINC-250k [18]. ChEMBL 21 comprises over one million chemical compounds that have been reported in biological assays. Meanwhile, the ZINC database is the largest chemical structure database and contains billions of chemical structures. The subset ZINC-250k, comprising 250k chemical structures, was used as a benchmark dataset for training generative models. The cheML.io dataset [9] was employed for the generated chemical structures, which were created using several generative models (Table 1).

**Table 1:** Datasets used for model development.

Dataset	Subcategory	ChEMBL 21	ZINC-250k	cheML.io	MOSES
**AE dataset**	**Training**	1388,488	-	-	-
	**Test**	347,123	-	-	-
Classification dataset* (D_**cls**_**)**	**Training**	-	131,319	452,023	-
	**Validation**	-	14,592	49,741	-
	**Test**	-	14,592	49,261	-
Ensemble dataset* * (D_**ens**_**)**	**Training**	-	72,611	82,275	-
	**Validation**	-	8069	9143	-
	**Test**	-	8069	9144	-
Demonstration dataset* **	**MOSES-test set** **(real)**	-	-	-	176,074
	**Generated**	-	-	-	719,999

*Based on the Tanimoto similarity (S_T_) of the ECFP4 fingerprint, chemical structures with moderate similarity (0.6 > S_T_ ≥ 0.3; cheML.io-based maximal S_T_ were calculated).

* * Structures not in training or validation of the classification dataset are used.

* ** Detailed information on the MOSES dataset is available in Table S1.

The chemical structures of each dataset were collected, and molecular properties, including QED [13], SAscore [14], logP, and MW, were calculated using the *RDKit* Python package. All chemical structures were encoded into a molecular fingerprint, ECFP4 [19], using *RDKit* before being further used for model training and testing.

To reconstruct the similarity-paired dataset based on cheML.io (Table 1), Tanimoto similarities between the chemical structures of ZINC-250k and cheML.io were calculated. The chemical structures of cheML.io were divided into two datasets based on their maximum similarity. First, the classification dataset (D_cls_) was selected to train the classification models. This dataset contained chemical structures with a maximal Tanimoto similarity ranging from 0.3 to 0.6. To identify the chemical structures of the ZINC-250k dataset as counterparts of cheML.io in the D_cls_, the chemical structure with the highest similarity to ZINC-250k was selected. For the ensemble dataset (D_ens_), chemical structures not used for training or validation of the classification models (D_cls_-training and D_cls_-validation, respectively) were used. The Tanimoto similarity of the ECFP4 fingerprint was utilized to measure the similarity between molecular fingerprints..

The demonstration dataset of Ertl and Schuffenhauer [14], containing 40 chemical structures with ChemistScore values, was used. For the Spearman and Pearson correlation analyses, the *spearmanr* and *pearsonr* functions of the Python package *scipy* [20] were employed. Additionally, the MOSES dataset [10] was applied (Table 1; Table S1) for model validation. The MOSES dataset is widely used and serves as a standardized platform for evaluating the performance of molecular generation models. It not only provides chemical structures generated by various models but also offers metrics to assess molecular properties, such as the SAscore. Specifically, the MOSES dataset contains the sample chemical structure datasets generated by eight different models [4], [7], [21], [22], [23], as well as the test dataset from ZINC. To ensure data integrity, any infeasible or duplicate structures were removed from the ∼90,000 generated chemical structures and the 176,074 real chemical structures. The feasibility of chemical structures was determined using the *RDKit* package, which applies SMILES to *RDKit* molecular objects.

The two-dimensional uniform manifold approximation and projection (UMAP) model was fitted with ECFP4-encoded chemical structures from the ZINC-250k and cheML.io datasets using the Python package *umap-learn*. To match the number of chemical structures in each dataset, the matched SMILES were used to fit the UMAP models.

### 2.2 Anomaly detection model

We used the *TensorFlow* package to construct an autoencoder (AE) as the anomaly detection model. To minimize the recovery loss for the test dataset, hyperparameters (number of nodes, hidden layers, and learning rate) for the AE model were optimized by a grid search (Table S2) using the ChEMBL21 training dataset. For loss calculation, binary cross-entropy was applied to each fingerprint component. The recovery scores of chemical structures were measured using the cosine similarities S_C_ between the real fingerprint vectors *x*_*f*_ and AE-recovered fingerprint vectors *x`*_*f*_, as represented by Eq. (1):

(1) $$S_{C}(x_{f},{x`}_{f})=\frac{x_{f}\cdot {x`}_{f}}{\left\|x_{f}\right\|\;\left\|{x\prime }_{f}\right\|}$$

### 2.3 Classification models

We tested classification models based on deep neural network (DNN), extreme gradient boosting (XGBoost), random forest (RF), and logistic regression (LR) models. D_cls_ (Table 1) was employed for classification model training by dividing it into training, validation, and test datasets at a 9:1:1 ratio.

The *TensorFlow* Python package was used to implement the DNN. The hyperparameters (architecture, dropout rate, and learning rate) for the DNN (Table S3) were optimized to minimize the binary cross-entropy loss for classification. The best model was selected with early stopping based on the area under the receiver operating characteristic (AUROC) curve score for the validation dataset.

The XGBoost classifier was trained using the *XGBoost* Python package. For optimization of the hyperparameters (number of estimators, subsample, subsample ratio of columns, maximal depth, minimal child weight, learning rate, and gamma), seven hyperparameters were distributed into four groups; each corresponding round of hyperparameter set was optimized. The best hyperparameter set was selected based on the AUROC validation set for each hyperparameter group round (Table S4).

RF and LR classifiers were trained using the *scikit-learn* package. The hyperparameters of RF (number of trees, depth, maximal leaf nodes, minimal sample split, and minimal sample leaf) were optimized by selecting the best hyperparameter set based on the AUROC for the validation dataset (Table S5). For the LR classifier, hyperparameters (penalty, solver, maximal number of iterations, and inverse of regularization strength) were also optimized (Table S6) as the identical method for RF. Finally, to select the best type of classification model among DNN, XGBoost, RF, and LR, the AUROC values of the validation dataset were assessed [24].

### 2.4 Ensemble model

For the ensemble model of AnoChem, the LR model of the *scikit-learn* package was used. The dataset for ensemble models (D_ens_, Table 1) was randomly divided into training, validation, and test datasets at a 9:1:1 ratio. In addition to the recovery score from the anomaly detection model and realistic probability from the classification model, molecular properties, namely SAscore, QED, MW, and logP, served as input features for the ensemble model. To ensure the best feature set for the ensemble model, LR models were trained with or without SAscore, QED, MW, and logP; the best set of features was selected (Table S7). The output value of the trained ensemble model (*P*_*Real*_) ranged from 0.0 to 1.0. To evaluate the performance of the models, the accuracy, AUROC, precision, recall, F1 score, and MCC were calculated.

### 2.5 Correlation analysis of molecular fingerprint bit co-occurrence

To analyze the correlation of molecular fingerprint bit co-occurrence, we counted the occurrence of molecular fingerprint bits across all chemical structures in ZINC-250k and cheML.io. We constructed contingency tables for bit-by-bit pairs from a combination of 12 bits, which exhibited a feature importance > 0.1 in the RF classification model. The expectation of co-occurrence was calculated by Eq. (2):

(2) $$p\left(B_{a}\right)\cdot p\left(B_{b}\right)\cdot n$$

where B_x_ refers to the occurrence of ECFP4 bit x, and n is the number of whole cases. To obtain the ratio of observed co-occurrence over expected, the number of co-occurred cases was divided by the co-occurrence expectation. For co-occurrence analysis, *X*^*2*^ and Fisher’s exact test were conducted using the *chi2_contingency* and *fisher exact* functions of the *scipy* module.

## 3 Results and discussion

### 3.1 Development of AnoChem

We developed AnoChem, a computational framework that predicts anomalies in chemical structures. The AnoChem framework functions through a three-stage prediction process (Fig. 1). The initial stage involves autoencoder models for anomaly prediction, while the second stage employs a classifier model to distinguish real compounds from generated compounds created by the model. In the final stage, the outcomes of the preceding stages are integrated using an ensemble model to deliver a prediction.

In the first stage, we utilized an AE-based anomaly detection approach to identify abnormal structural features at the molecular fingerprint level. If the AE models trained with the molecular fingerprints of real compounds failed to accurately reproduce the molecular fingerprints of the input compound, they were considered less likely to be real structures. To achieve more comprehensive coverage of real compounds and include those generated by generative models, we obtained real compound structures from the ChEMBL database—one of the most extensive manually curated databases of bioactive molecules—and the ZINC-250k dataset—the most commonly adopted benchmark for training molecular generative models (Table 1). We successfully trained the AE model employing the ECFP4 fingerprint without overfitting. The test loss value for the AE model based on the ECFP4 was 0.018.

In the second stage, we constructed four machine learning models based on DNN, XGBoost, RF, and LR algorithms and selected the best classification model. To improve model robustness and avoid overfitting, we compiled a dataset of compounds exhibiting moderate Tanimoto similarity to real ones (0.6 > S_T_ ≥ 0.3; Table 1 and Table 2). We reasoned that including compounds with either extremely low or high similarity could either oversimplify differentiation or hinder accurate distinction. Upon model training, the XGBoost model achieved a superior AUROC score of 0.967 (ACC = 0.918; MCC = 0.757) compared with the other classification models (Fig. S2). Hence, the XGBoost model was selected as the classification model for AnoChem.

**Table 2:** Performance of AnoChem ensemble and classification model.

	Ensemble (LR)	Classification (XGBoost)	Number of chemical structures
**Accuracy**	0.825	0.804	17,213
**Accuracy (ZINC-250k)**	0.796	0.763	8069
**Accuracy (cheML.io)**	0.851	0.839	9144
**AUROC**	0.900	0.890	
**Precision**	0.825	0.807	
**Recall**	0.796	0.763	
**F1 score**	0.810	0.785	
**MCC**	0.649	0.606	

LR, logistic regression; XGBoost, extreme gradient boosting; AUROC, area under the receiver operating characteristic curve; MCC, Matthew’s correlation coefficient.

**Fig. 2:**
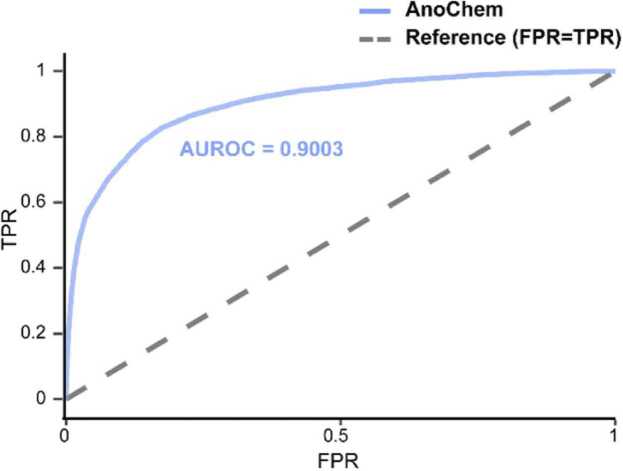
Performance of AnoChem. The AnoChem ensemble model has an AUROC of 0.900 for classifying real (ZINC-250k) and generated (cheML.io) chemical structures. The sub-score, *P*_*Real, XGB*_—result of the XGBoost classification model—has an AUROC of 0.890 on the identical test.

In the final stage, we constructed an ensemble model based on LR to determine the final probability score *P*_*Real*_ for classifying the molecules as real or generated (see Materials and methods). The ensemble model was trained using predictive scores from the anomaly-detection model and the XGBoost classification model using subsets of the ZINC-250k and cheML.io datasets (Table 1) that were not utilized for training the classification models. To achieve better performance, we tested four molecular properties: SAscore, QED, MW, and logP, to be additionally used for the ensemble model. The feature set with QED and MW showed the best performance in the validation AUROC (Table S7). Meanwhile, usage of SAscore does not guarantee increased AUROC in validation, with decreases observed in some cases (Table S7). This will be further investigated in Section 3.2. The AnoChem platform exhibited an overall performance with an AUROC value of 0.900 (Fig. 2; Table 2). The XGBoost classification sub-model achieved an AUROC value of 0.890.

Although the AnoChem achieved high prediction performance, we did not observe significant differences in the molecular properties or chemical structures between the real and generated compounds. Specifically, there was no notable disparity in the molecular properties (QED, SAscore, logP, and MW) of the chemical structures (Fig. S1A–D). The chemical structures in the cheML.io dataset exhibited a slightly higher SAscore distribution than those in the ZINC-250k dataset; however, this difference was negligible. Additionally, no apparent structural differences were observed between the compounds based on the ECFP4 embedding of their chemical structures (Fig. S1E). Overall, these findings indicate that there was no significant disparity in the chemical characteristics between real and generated compounds. However, there appear to be discernible subtle characteristics that differentiate the groups, which might be utilized for their classification.

### 3.2 Comparative analysis of AnoChem prediction results with SAscore and ChemistScore

**Fig. 3:**
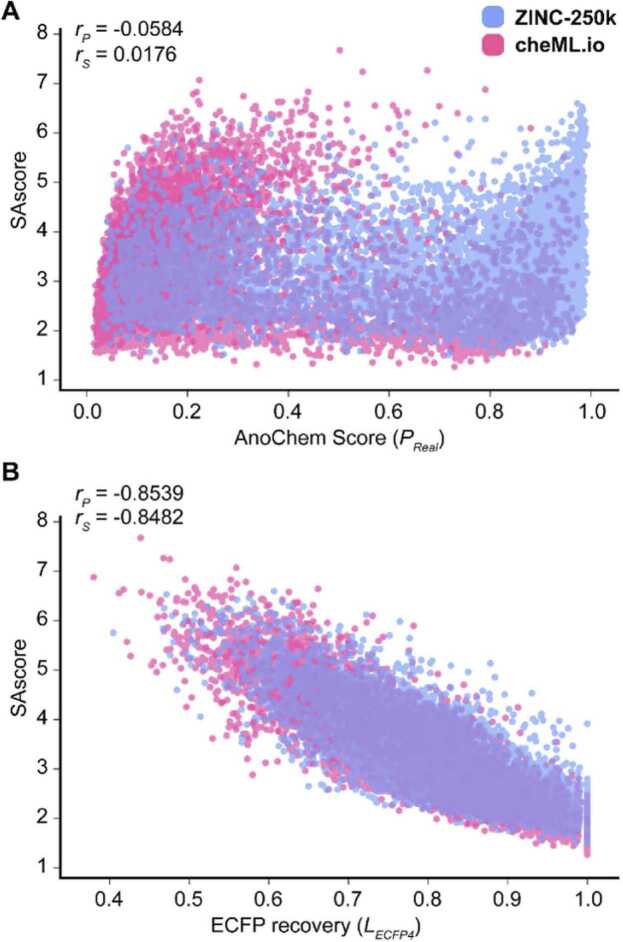
Comparison of AnoChem score and SAscore. (A) AnoChem score and SAscore are unrelated (Pearson correlation coefficient *r*_*P*_ = −0.058; Spearman’s rank correlation coefficient *r*_*S*_ = 0.018). (B) The ECFP4-based recovery score and SAscore are negatively correlated (*r*_*P*_ = −0.854; *r*_*S*_ = −0.848).

To further validate the prediction results of AnoChem, we compared the scores of each model with the SAscore and ChemistScore using the test datasets of the ensemble datasets (Table 1) and the demonstration dataset of Ertl and Schuffenhauer [14]. The SAscore is the synthetic accessibility with the combined properties of the fragments of the chemical structure with a penalty of complexity, and the ChemistScore represents the average of manual scores assigned by nine medicinal chemists, indicating the synthesis difficulty of the compounds. Owing to the distinct objectives of the anomaly detection and classification model, the scores of each model were not closely related to each other. Furthermore, in the relationship between AnoChem and SAscore, no significant correlation was detected between *P*_*Real*_ and SAscore (Fig. 3A; Pearson correlation coefficient, *r*_*P*_ = −0.0584; Spearman’s rank correlation coefficient, *r*_*S*_ = 0.0176). Notably, while the AnoChem score distinguishes whether a compound is real or generated by a model, it does not reflect the level of chemical synthesis difficulty, such as synthetic accessibility. However, there was a strong correlation between the SAscore and the ECFP4-based anomaly detection model score *L*_*ECFP4*_ (Fig. 3B; *r*_*P*_ = −0.8539; *r*_*S*_ = −0.8482). Furthermore, *L*_*ECFP4*_ expression was highly correlated with SAscore and ChemistScore (Fig. 3B; Fig. S3).

These results explain why using SAscore in the AnoChem ensemble model decreases the classification performance (Table S7): the usage of SAscore duplicates the molecular information, which has already been inferred by *L*_*ECFP4*_. However, the classification performance of *L*_*ECFP4*_ was superior to that of SAscore, which correlates with the feature selection results for the ensemble model (Table S7).

### 3.3 Investigation of feature importance of molecular fingerprint bits

To investigate the structural signature of the generated structures, the feature importance of the RF classification was further analyzed. Specific molecular fingerprint bits, particularly bit 1171, demonstrated a high level of importance (Fig. S4; Table S8). Moreover, the top 12 bits exhibited importance values > 0.01, while the top 56 bits accounted for a cumulative feature importance exceeding 0.5, with 588 bits surpassing 0.9. These results suggest that certain bit signatures are specific to the generated chemical structures.

According to the ECFP4 encryption mechanism, each bit is not expected to have independent structural features. Rather, bits containing important fragments may exhibit strong correlations. To confirm this, the *X*^2^ and Fisher’s exact tests were performed on the top 12 bits across all chemical structures of the ZINC-250k and cheML.io datasets (Materials and methods). The co-occurrence analysis results confirmed that certain bit pairs had clear co-occurrence tendencies (Fig. S5; Table S9). For example, among the top 12 bits, the bit 1243 and 1911 pair showed 4.43-fold higher observation of co-occurrence than the expectation.

### 3.4 Demonstration of AnoChem on the MOSES dataset

**Fig. 4:**
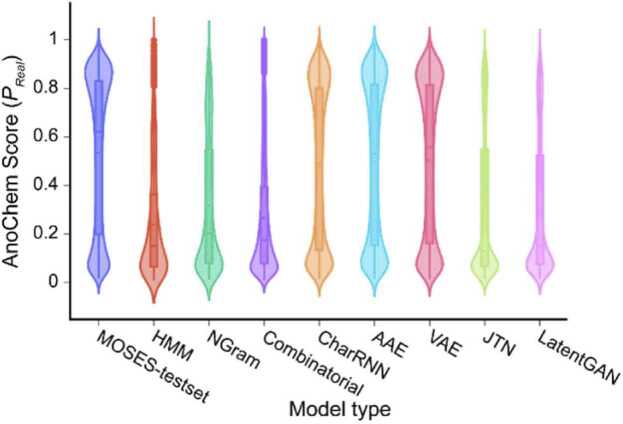
AnoChem application on generated chemical structures using benchmark models. AnoChem scores evaluated for MOSES sample datasets (https://github.com/molecularsets/moses), including refined ZINC data (MOSES-test set) and generated chemical structures using HMM, NGram, Combinatorial, CharRNN, AAE, VAE, JTN and LatentGAN. AnoChem predicted 57.5% of MOSES-test set as real structures. For generated structures, the prediction rates of real ranged from 16.3 to 53.4%. Abbreviations: ROC, receiver operating characteristic; AUROC, area under ROC.

To demonstrate the capabilities of AnoChem using an external dataset, we utilized the MOSES [10] sample dataset, which contains refined chemical structures from the ZINC dataset, and generated chemical structures using eight different generative models (Table S1; see Materials and methods). We analyzed the chemical structures of the MOSES-test set (i.e., the test dataset of the refined ZINC dataset) and the generated chemical structures of the generative models using AnoChem (Fig. 4; Fig. S6; Table S1). AnoChem predicted that approximately 57.5% of the molecules in the MOSES-test set were real (Fig. 4; Table S1; *P*_*Real*_ = 0.535 ± 0.316, for mean ± SD which are from the ZINC database. For the generated molecules, VAE had the highest prediction rate for *P*_*Real*_ (*P*_*Real*_ = 0.504 ± 0.318), with 53.4% of the molecules classified as real by AnoChem. AAE and CharRNN [25] had real prediction rates of 51.8% and 49.5%, respectively (0.497 ± 0.322 and 0.477 ± 0.322 for mean ± SD of *P*_*Real*_, respectively). The evaluation results using AnoChem correlated with those using similarity to the nearest neighbor (SNN) and scaffold similarity, confirming the competitive performance of AAE, CharRNN, and VAE [10]. Moreover, the AnoChem score correlates with scaffold-based similarity tests, such as SNN and scaffold similarity. Further, in terms of FCD, in which CharRNN and VAE showed the best performance over other model types, the AnoChem score of those models showed the third and first highest score among the generative models. In contrast, for the hidden Markov model (HMM), NGram, and Combinatorial, all score metrics (AnoChem results, SNN, scaffold similarity, and FCD) exhibited poor results, and the distributions of the SAscore and *L*_*ECFP4*_ for the generated molecules of those models were easily distinguishable from those of the real molecules (Fig. S6). In summary, using AnoChem, we found that 72.3% of the molecules examined (337,631 generated and 182,944 invalid out of 719,999) were invalid or generated. Meanwhile, approximately 57.5% of the molecules in the MOSES test set were predicted to be real (Table S1). These results suggest that AnoChem can distinguish between the putatively generated chemical structures and real structures and also examine the performance of generative models.

Although AnoChem demonstrated competitive performance on various metrics for detecting generated molecules, other metrics should be considered in conjunction with AnoChem to achieve more robust computational drug screening. The complementary use of other metrics, such as SAscore, is expected to reduce the number of false positive predictions.

## 4 Conclusion

In this study, we present AnoChem as a method to predict anomalies in the chemical structures of input compounds. AnoChem can accurately distinguish between generated and real compounds, even when the generated chemical structures have molecular properties similar to those of real compounds. In the demonstration on the cheML.io and MOSES datasets, AnoChem showed competitive performance on other metrics for detecting generated molecules, such as SAscore. Furthermore, several generative models of the MOSES dataset were examined by applying AnoChem to the generated chemical structures, and the performance test results using AnoChem were correlated with FCD, SNN, and scaffold similarity. Additionally, the assessment of AnoChem with an external dataset revealed that it detected 72.3% of the generated molecules. Taken together, AnoChem could be a promising tool for filtering invalid molecules during the early stages of drug discovery as well as for fine-tuning generative models for *de novo* molecules.

## Author statement

J.Y.R. conceived and supervised the study. C.G., and J.Y.R. developed the models, C.G., W.D.J., K.S.O. and J.Y.R analyzed the data. C.G., W.D.J., K.S.O., and J.Y.R. wrote the manuscript. All authors have read and agreed to the published version of the manuscript.

## CRediT authorship contribution statement

**Woo Dae Jang:** Data curation, Investigation, Writing – original draft, Writing – review & editing. **Changdai Gu:** Conceptualization, Data curation, Methodology, Visualization, Writing – original draft, Writing – review & editing. **Jae Yong Ryu:** Conceptualization, Data curation, Funding acquisition, Investigation, Methodology, Validation, Visualization, Writing – original draft, Writing – review & editing. **Kwang-Seok Oh:** Data curation, Validation, Writing – original draft, Writing – review & editing.

## Declaration of Competing Interest

The authors declare that they have no known competing financial interests or personal relationships that could have appeared to influence the work reported in this paper.

## Data Availability

The source code for AnoChem is available at https://github.com/CSB-L/AnoChem.

## Footnotes

Appendix A Supplementary data associated with this article can be found in the online version at doi:10.1016/j.csbj.2024.05.017.
