# Supplementary material for: AnoChem: Prediction of chemical structural abnormalities based on machine learning models
Source: Comput Struct Biotechnol J. 2024 May 15;23:2116–21. doi: 10.1016/j.csbj.2024.05.017 (PMC11130677; doi:10.1016/j.csbj.2024.05.017)
Supplement: Supplementary file 1 — Supplementary material [file mmc1.docx]

**Supplementary data**

**AnoChem: Prediction of chemical structural abnormalities based on machine learning models**

Changdai Gu^a,b^, Woo Dae Jang^c,d^, Kwang-Seok Oh^c,d^ and Jae Yong Ryu^a,e,*^

*^a^ Artificial Intelligence Laboratory, Oncocross Co., Ltd., Saechang-ro, Mapo-gu, Seoul, 04168, Republic of Korea*

*^b^ Department of Artificial Intelligence, College of Computing, Yonsei University, 50 Yonsei-ro, Seodaemun-gu, Seoul, 03722, Republic of Korea*

*^c^ Data Convergence Drug Research Center, Korea Research Institute of Chemical Technology, 141 Gajeong-ro, Yuseong-gu, Daejeon, 34114, Republic of Korea*

*^d^ Department of Medicinal and Pharmaceutical Chemistry, University of Science and Technology, Daejeon, 34129, Republic of Korea*

*^e^ Department of Biotechnology, Duksung Women’s University, 33 Samyang-Ro 144-Gil, Dobong-gu, Seoul, 01369, Republic of Korea*

⁎ Corresponding authors.

*E-mail address*: jyryu@duksung.ac.kr (J.Y. Ryu)

## **Supplementary Figures**

##
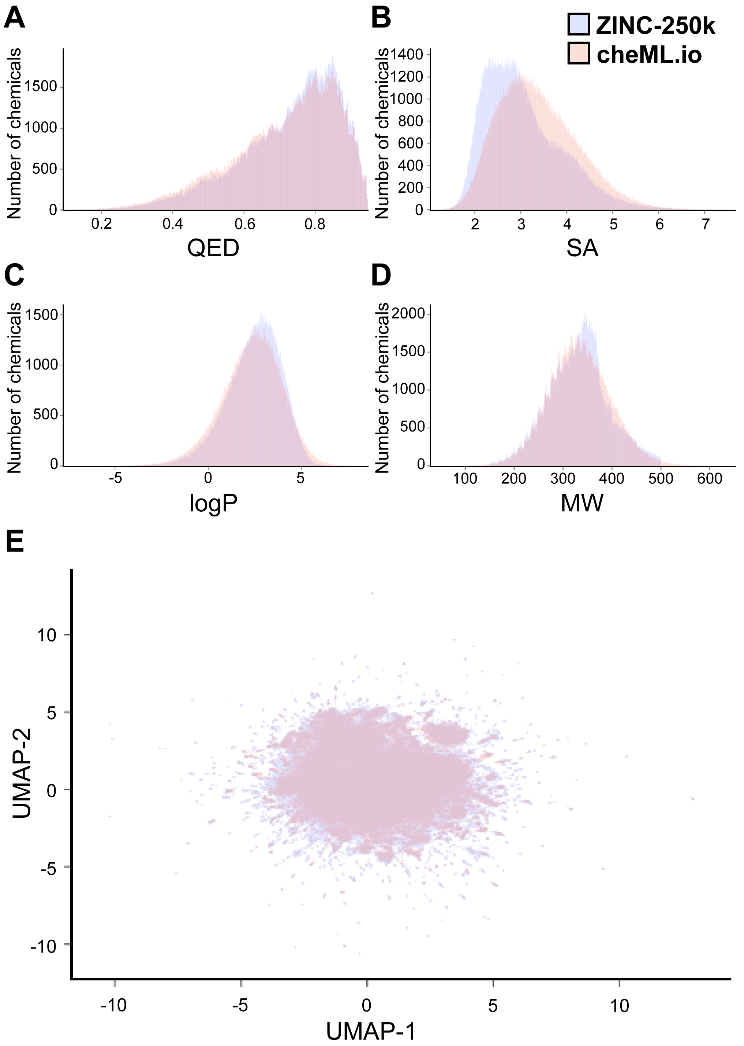


**Fig. S1.** Comparison of molecular properties between real and generated chemical structures. This comparison involves 249,455 chemical structures (i.e., "real compounds") from the ZINC-250k dataset and an equivalent number of randomly selected structures (i.e., "generated compounds") from the cheML.io dataset (Table 1). Four molecular properties are compared: (A) QED, (B) SAscore, (C) logP, and (D) MW. (E) Real and generated chemical structures are encoded and analyzed as the ECFP4 fingerprint. The UMAP embedding plot reveals no specific distribution pattern for the entire dataset of real (ZINC-250k) and generated structures (cheML.io). Abbreviations: QED, Quantitative Estimate of Druglikeness; SAscore, synthetic accessibility score; MW, molecular weight; UMAP: uniform manifold approximation and projection.


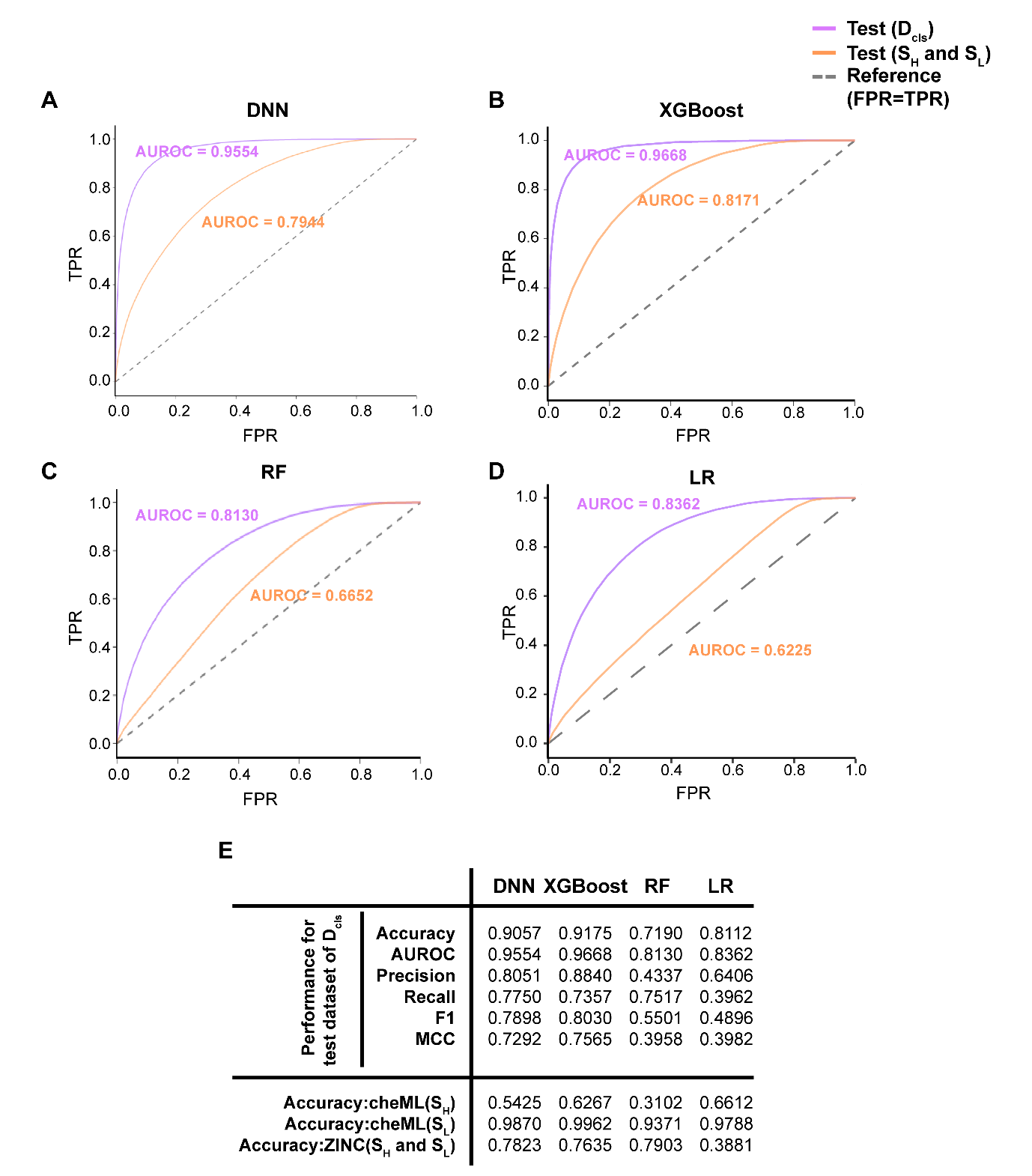
**Fig. S2.** Training results of DNN, XGBoost, RF, and LR classification models of AnoChem. ROC for (A) DNN, (B) XGBoost, (C) RF, and (D) LR classifier using test dataset of moderate similarity (D_cls_) and high and low similarity (S_H_ and S_L_; these are the remaining chemical structures excluded from D_cls_) pairs of ZINC-250k dataset [1] and cheML.io dataset [2]. (E) Overall performance of DNN, XGBoost, RF and LR classifier were measured. Abbreviations: DNN, deep neural network; XGBoost, eXtreme gradient boosting; RF, random forest; LR, logistic regression; ROC, receiver operating characteristic; AUROC, area under ROC; MCC, Matthews correlation coefficient.


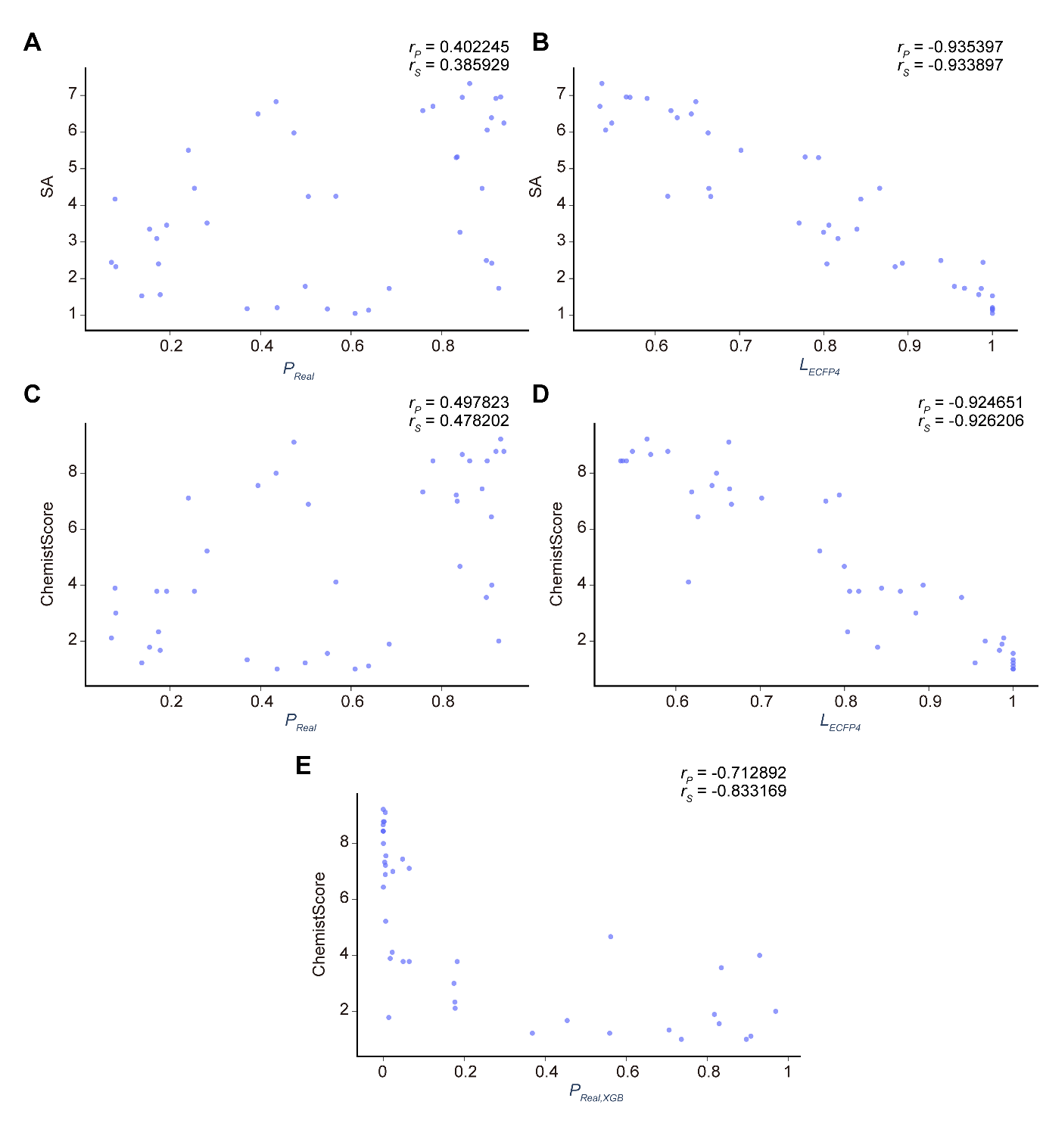
**Fig. S3.** Correlation of SAscore or ChemistScore with AnoChem result scores. (A) Correlation analysis between SAscore and AnoChem final score *P_Real_*, the correlation coefficient is relatively weaker than (B) between SAscore and *L_ECFP4_* (sub-score of AnoChem). Correlation for ChemistScore, (C) *P_Real_* showed the weakest correlation (D, E). A demonstrational dataset of *Ertl and Schuffenhauer* [3], containing ChemistScore for 40 chemical structures, was used. (*P_Real_*, realistic probability score; *L_ECFP4_*, recovery score for ECFP4; SA, Synthetic accessibility score; *r_P_*, Pearson correlation coefficient; *r_S_*, Spearman’s rank correlation coefficient)


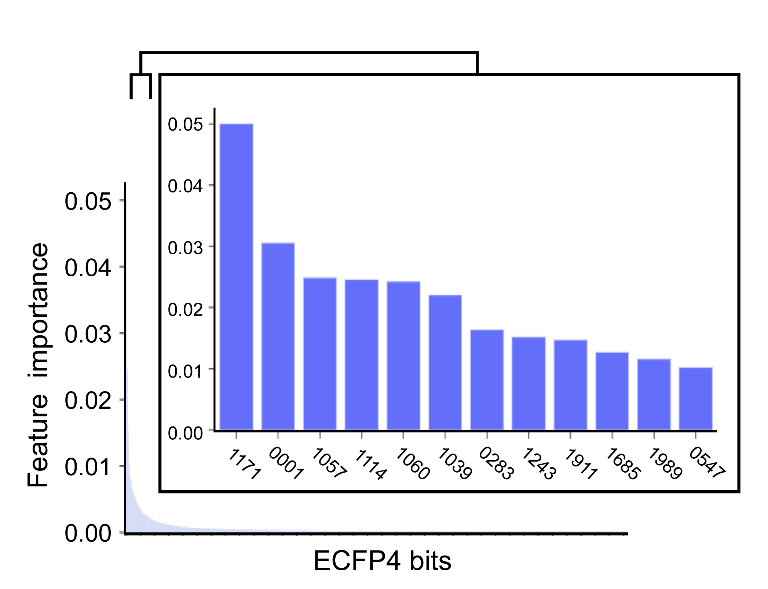


**Fig. S4.** Feature importance of RF classifier. ECFP4 [4] bit 1171 has the highest feature importance. Twelve ECFP4 bits among 2,048 bits exceed 0.1 for the feature importance of the RF classifier. The 56 ECFP4 bits with the highest feature importance exhibit a cumulative feature importance > 0.5 and 588 bits > 0.9.


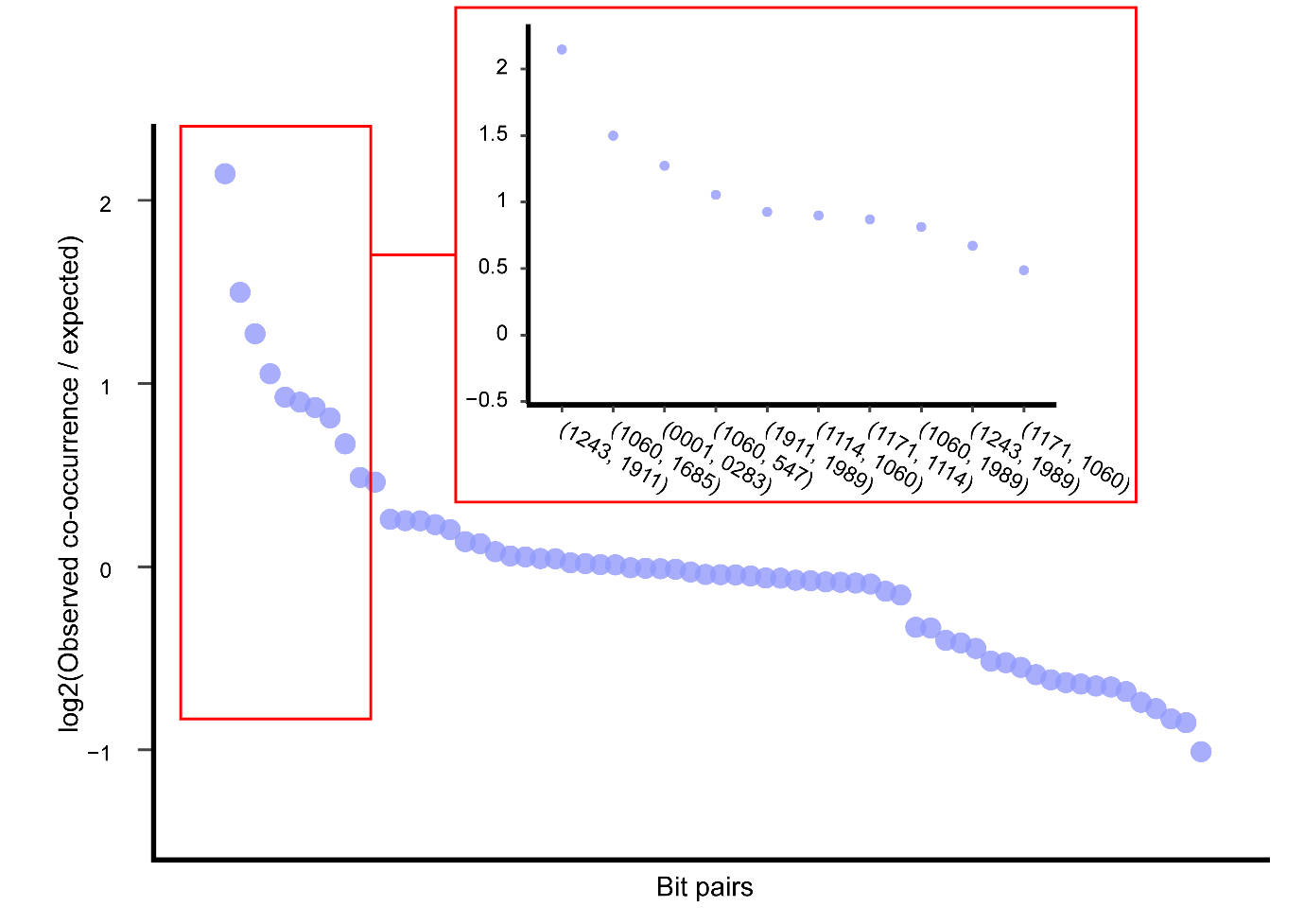


**Fig. S5.** Co-occurrence of ECFP4 bit pairs for ZINC-250k and cheML.io molecular datasets. Co-occurrence of 12 ECFP4 bits is analyzed; feature importance in the Random Forest classification model for ZINC-250k and cheML.io classification task exceeds 0.1. Observed co-occurred cases over expected are depicted in logarithmic scale; 27 of the 66 pairs exceed 0.0 and 39 are below 0.0 (log_2_(observed/expected) = 1.0941 ± 0.6050, for mean ± SD).


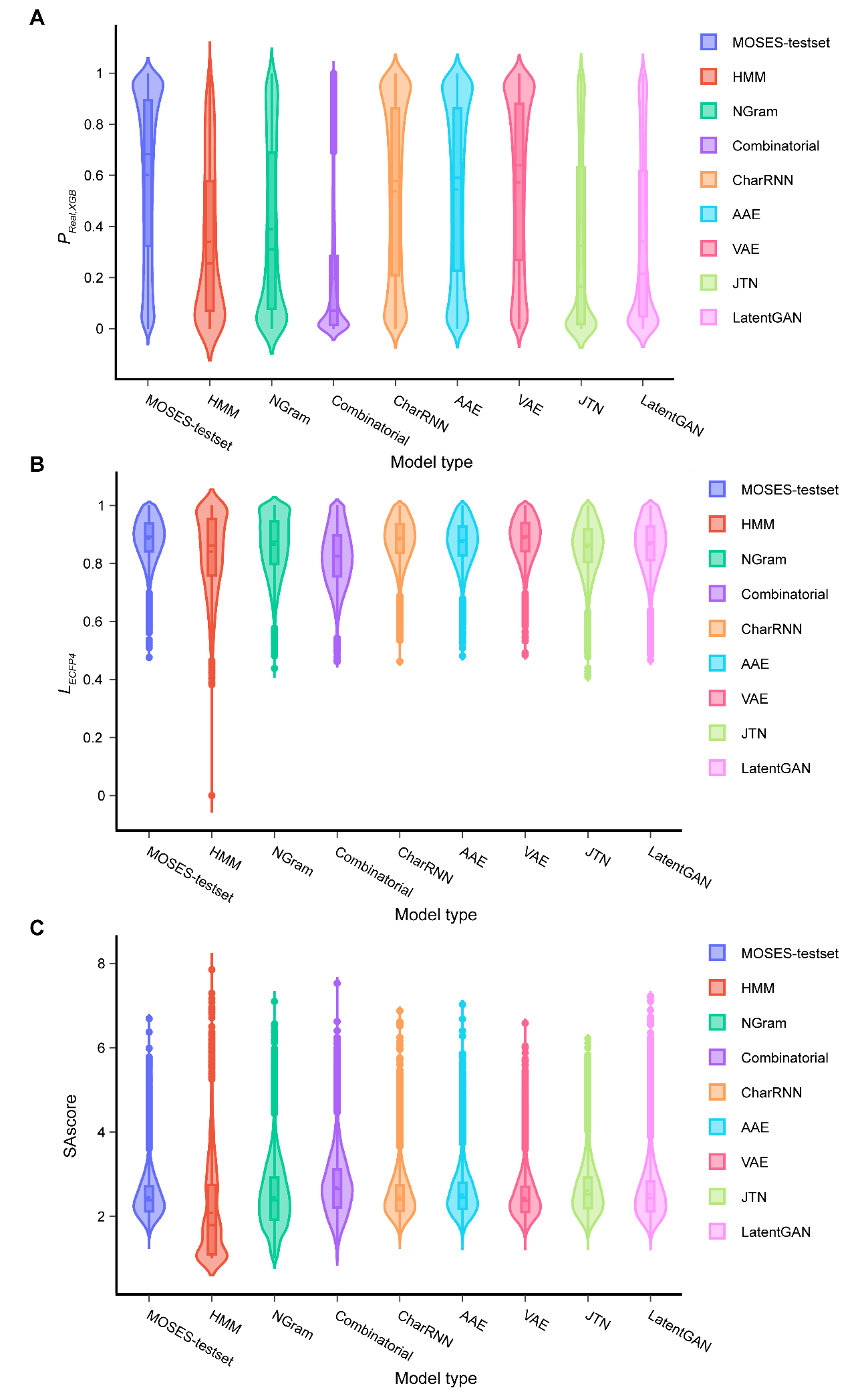


**Fig. S6.** Analysis results of MOSES sample dataset using AnoChem. Using AnoChem sub-models, (A) *P_Real,XGB_* and (B) *L_ECFP4_* were obtained for MOSES sample datasets [5], composed of real chemical structures (MOSES-test set) from the ZINC database [1]; chemical structures were generated for each generative model. (C) SAscore of the dataset. For *P_Real,XGB_*, the dataset for the real chemical structure, the MOSES-test set has the highest score on average compared with all other molecular pools generated by models. Molecules generated by CharRNN, AAE [6], and VAE [7] have distributions with the highest average *P_Real,XGB_*, which are similar to the real molecules. *L_ECFP4_*, a subscore of the AnoChem platform that correlates with the (C) SAscore and has a similar distribution as the SAscore counterparts (higher scores indicate that more molecular structures can be recovered). In both the distributions of SAscore and *L_ECFP4_*, the distribution of chemical structure scores from generative models is similar to that of real molecules, excluding HMM, NGram, and Combinatorial. The used datasets are accessible at https://github.com/molecularsets/moses.

## **Supplementary Tables**

**Table S1. Model performance on the MOSES dataset**

| **Category** | **Property** | **MOSES-test dataset** | **HMM** | **NGram** | **Combinatorial** | **CharRNN [8]** | **AAE [6]** | **VAE [7]** | **JTN [9]** | **LatentGAN [10]** |
| --- | --- | --- | --- | --- | --- | --- | --- | --- | --- | --- |
| Input | Input SMILES | 176,074 | 90,000 | 90,000 | 90,000 | 90,000 | 90,000 | 90,000 | 89,999 | 90,000 |
| Structural feasibility | Valid SMILES* | 176,074 | 3,253 | 18,164 | 87,391 | 87,375 | 83,584 | 87,122 | 89,686 | 80,480 |
|  | Invalid SMILES* | 0 | 86,747 | 71,836 | 2,609 | 2,625 | 6,416 | 2,878 | 313 | 9,520 |
|  | Invalid SMILES rate | 0 | 0.9639 | 0.7982 | 0.029 | 0.0292 | 0.0713 | 0.032 | 0.0035 | 0.1058 |
| AnoChem | n(P≥0.5) | 101,304 | 530 | 5,036 | 15,773 | 43,281 | 43,334 | 46,537 | 24,027 | 20,906 |
|  | n(P<0.5) | 74,770 | 2,723 | 13,128 | 71,618 | 44,094 | 40,250 | 40,585 | 65,659 | 59,574 |
|  | P>0.5 :ratio | 0.5753 | 0.1629 | 0.2773 | 0.1805 | 0.4953 | 0.5184 | 0.5342 | 0.2679 | 0.2598 |
|  | P<0.5 :ratio | 0.4247 | 0.8371 | 0.7227 | 0.8195 | 0.5047 | 0.4816 | 0.4658 | 0.7321 | 0.7402 |
|  | (P<0.5) or invalid :ratio | 0.4247 | 0.9941 | 0.944 | 0.8247 | 0.5191 | 0.5185 | 0.4829 | 0.733 | 0.7677 |
|  | mean | 0.5349 | 0.2388 | 0.3171 | 0.2667 | 0.4766 | 0.4973 | 0.504 | 0.2989 | 0.3006 |
|  | SD | 0.316 | 0.2224 | 0.2755 | 0.2396 | 0.3219 | 0.3215 | 0.3183 | 0.3 | 0.2836 |
|  | min | 0.0171 | 0.0104 | 0.0114 | 0.0104 | 0.0143 | 0.0147 | 0.0149 | 0.0152 | 0.0155 |
|  | Q1 | 0.1993 | 0.0650 | 0.0804 | 0.0790 | 0.1334 | 0.1537 | 0.1617 | 0.0672 | 0.0761 |
|  | median | 0.6216 | 0.1504 | 0.2026 | 0.1748 | 0.4917 | 0.5320 | 0.5583 | 0.1287 | 0.1536 |
|  | Q3 | 0.8307 | 0.3622 | 0.5467 | 0.3930 | 0.8015 | 0.8163 | 0.8140 | 0.5501 | 0.5239 |
|  | max | 0.9841 | 0.9982 | 0.9936 | 0.9992 | 0.9810 | 0.9837 | 0.9791 | 0.9746 | 0.9824 |
| QED | mean | 0.8066 | 0.5439 | 0.718 | 0.6571 | 0.8044 | 0.8007 | 0.807 | 0.805 | 0.8018 |
|  | SD | 0.0949 | 0.1051 | 0.1294 | 0.1821 | 0.0956 | 0.0976 | 0.0952 | 0.0988 | 0.0982 |
| SA | mean | 2.4496 | 2.0811 | 2.452 | 2.6892 | 2.4657 | 2.5158 | 2.4365 | 2.6089 | 2.5206 |
|  | SD | 0.4598 | 1.0884 | 0.7424 | 0.6701 | 0.4717 | 0.4808 | 0.4636 | 0.5739 | 0.5563 |
| logP | mean | 2.439 | 1.6767 | 2.1409 | 3.0167 | 2.4399 | 2.4677 | 2.447 | 2.4241 | 2.4721 |
|  | SD | 0.9267 | 0.924 | 1.125 | 1.798 | 0.9888 | 0.9941 | 0.9494 | 1.0099 | 1.0083 |
| MW | mean | 307.2344 | 133.0652 | 243.0159 | 334.331 | 308.2281 | 315.7897 | 306.2253 | 306.6823 | 306.8287 |
|  | SD | 28.0841 | 60.0709 | 84.3245 | 112.3383 | 30.1607 | 32.8591 | 28.4378 | 28.8858 | 34.4392 |

* Infeasible or duplicated SMILES are excluded

**Table S2. Hyperparameter optimization results for Autoencoder models**

|  |  | **Considered parameters** | **Best parameter** |
| --- | --- | --- | --- |
| Hyperparameter | Node | 32; 64 | 64 |
|  | Hidden layers | 1; 2; 3 | 3 |
|  | Learning rate | 0.001; 0.005; 0.01; 0.05 | 0.01 |
| Performance of the best hyperparameter | Loss | Train | 0.0180 |
|  | Loss | Test | 0.0178 |

**Table S3. Hyperparameter optimization results for DNN-classifier**

|  |  | **Considered parameters** | **Best parameter** |
| --- | --- | --- | --- |
| **Hyperparameter** | Nodes | 2048__512__256__128__32__1 2048__512__128__1 2048__512__32__1 2048__256__128__64__1 2048__256__64__16__1 | 2048__512__128__1 |
|  | dropout | 0.2; 0.4; 0.5 | 0.4 |
|  | learning_rate | 0.00001; 0.0001; 0.001 | 0.00001 |
| **Performance** | **Train** | **Accuracy** | 0.9778 |
|  |  | **AUROC** | 0.9962 |
|  |  | **Precision** | 0.9448 |
|  |  | **Recall** | 0.9572 |
|  |  | **F1 score** | 0.9509 |
|  |  | **MCC** | 0.9366 |
|  | **Validation** | **Accuracy** | 0.9068 |
|  |  | **AUROC** | 0.9557 |
|  |  | **Precision** | 0.8090 |
|  |  | **Recall** | 0.7713 |
|  |  | **F1 score** | 0.7897 |
|  |  | **MCC** | 0.7303 |

**Table S4. Hyperparameter optimization results for XGBoost-classifier**

|  | **Target hyperparams** | **Considered parameters** | **Best parameter** |
| --- | --- | --- | --- |
| **Hyperparameter** | n_estimators | 30; 50; 100; 150; 200 | 200 |
|  | subsample | 0.7; 0.8; 0.9; 1 | 1 |
|  | colsample_bytree | 0.7; 0.8; 0.9; 1 | 0.7 |
|  | max_depth | 2; 3; 5; 10; 15; 20; 30 | 30 |
|  | min_child_weight | 1; 3; 5 | 1 |
|  | eta | 0.01; 0.05; 0.1; 0.2; 0.3 | 0.2 |
|  | gamma | 0.1; 0.2; 0.3; 0.4; 0.5 | 0.1 |
| **Performance** | **Train** | **Accuracy** | 0.9995 |
|  |  | **AUROC** | 1.0000 |
|  |  | **Precision** | 0.9985 |
|  |  | **Recall** | 0.9993 |
|  |  | **F1 score** | 0.9989 |
|  |  | **MCC** | 0.9986 |
|  | **Validation** | **Accuracy** | 0.9183 |
|  |  | **AUROC** | 0.9667 |
|  |  | **Precision** | 0.8904 |
|  |  | **Recall** | 0.7297 |
|  |  | **F1 score** | 0.8021 |
|  |  | **MCC** | 0.7572 |

**Table S5. Hyperparameter optimization results for RF-classifier**

|  |  | **Considered parameters** | **Best parameter** |
| --- | --- | --- | --- |
| **Hyperparameter** | n_estimators | 50; 100; 200; 400 | 200 |
|  | max_depth | 10; 30; 50; 100 | 30 |
|  | max_leaf_nodes | 20; 50; 100; 200 | 200 |
|  | min_samples_split | 2; 5; 10 | 10 |
|  | min_samples_leaf | 1; 2; 3 | 1 |
| **Performance** | **Train** | **Accuracy** | 0.6989 |
|  |  | **AUROC** | 0.7926 |
|  |  | **Precision** | 0.4076 |
|  |  | **Recall** | 0.7447 |
|  |  | **F1 score** | 0.5268 |
|  |  | **MCC** | 0.3652 |
|  | **Validation** | **Accuracy** | 0.6968 |
|  |  | **AUROC** | 0.7902 |
|  |  | **Precision** | 0.4076 |
|  |  | **Recall** | 0.7425 |
|  |  | **F1 score** | 0.5263 |
|  |  | **MCC** | 0.3623 |

**Table S6. Hyperparameter optimization results for Logistic regression classifier**

|  |  | **Considered parameters** | **Best parameter** |
| --- | --- | --- | --- |
| **Hyperparameter** | penalty | l1; l2; elasticnet; None | l1 |
|  | solver | lbfgs; liblinear; newton-cg; newton-cholesky; sag; saga | saga |
|  | max_iter | 100; 200; 500; 1000 | 200 |
|  | C | 0.001; 0.01; 0.1; 1 | 1 |
| **Performance** | **Train** | **Accuracy** | 0.8147 |
|  |  | **AUROC** | 0.8392 |
|  |  | **Precision** | 0.6429 |
|  |  | **Recall** | 0.3975 |
|  |  | **F1 score** | 0.4912 |
|  |  | **MCC** | 0.4022 |
|  | **Validation** | **Accuracy** | 0.8143 |
|  |  | **AUROC** | 0.8382 |
|  |  | **Precision** | 0.6455 |
|  |  | **Recall** | 0.4021 |
|  |  | **F1 score** | 0.4955 |
|  |  | **MCC** | 0.4055 |

**Table S7. Feature selection results for ensemble model**

| **Features*** | **Validation** | | | | | |
| --- | --- | --- | --- | --- | --- | --- |
|  | **Accuracy** | **AUROC** | **Precision** | **Recall** | **F1 score** | **MCC** |
| QED_MW_ECFP4_XGBoost_cls_prob | 0.8215 | 0.8938 | 0.8267 | 0.7834 | 0.8045 | 0.6412 |
| SA_QED_MW_ECFP4_XGBoost_cls_prob | 0.8197 | 0.8936 | 0.8245 | 0.7818 | 0.8025 | 0.6376 |
| SA_QED_MW_LogP_ECFP4_XGBoost_cls_prob | 0.8190 | 0.8936 | 0.8241 | 0.7804 | 0.8017 | 0.6362 |
| QED_MW_LogP_ECFP4_XGBoost_cls_prob | 0.8204 | 0.8934 | 0.8251 | 0.7827 | 0.8034 | 0.6390 |
| SA_MW_LogP_ECFP4_XGBoost_cls_prob | 0.8189 | 0.8927 | 0.8249 | 0.7790 | 0.8013 | 0.6361 |
| MW_LogP_ECFP4_XGBoost_cls_prob | 0.8193 | 0.8926 | 0.8247 | 0.7805 | 0.8020 | 0.6369 |
| MW_ECFP4_XGBoost_cls_prob | 0.8193 | 0.8926 | 0.8248 | 0.7804 | 0.8020 | 0.6369 |
| SA_MW_ECFP4_XGBoost_cls_prob | 0.8194 | 0.8925 | 0.8254 | 0.7797 | 0.8019 | 0.6370 |
| SA_QED_MW_LogP_XGBoost_cls_prob | 0.8174 | 0.8920 | 0.8217 | 0.7797 | 0.8001 | 0.6330 |
| SA_QED_MW_XGBoost_cls_prob | 0.8173 | 0.8920 | 0.8213 | 0.7799 | 0.8001 | 0.6328 |
| SA_QED_LogP_ECFP4_XGBoost_cls_prob | 0.8181 | 0.8912 | 0.8240 | 0.7782 | 0.8004 | 0.6344 |
| QED_LogP_ECFP4_XGBoost_cls_prob | 0.8187 | 0.8909 | 0.8246 | 0.7790 | 0.8012 | 0.6357 |
| SA_QED_ECFP4_XGBoost_cls_prob | 0.8168 | 0.8906 | 0.8217 | 0.7780 | 0.7993 | 0.6318 |
| QED_ECFP4_XGBoost_cls_prob | 0.8172 | 0.8905 | 0.8219 | 0.7789 | 0.7998 | 0.6327 |
| SA_MW_XGBoost_cls_prob | 0.8159 | 0.8905 | 0.8205 | 0.7775 | 0.7984 | 0.6301 |
| SA_MW_LogP_XGBoost_cls_prob | 0.8161 | 0.8905 | 0.8208 | 0.7775 | 0.7986 | 0.6304 |
| SA_QED_LogP_XGBoost_cls_prob | 0.8146 | 0.8897 | 0.8199 | 0.7747 | 0.7967 | 0.6274 |
| SA_QED_XGBoost_cls_prob | 0.8122 | 0.8892 | 0.8170 | 0.7725 | 0.7941 | 0.6226 |
| SA_LogP_ECFP4_XGBoost_cls_prob | 0.8143 | 0.8883 | 0.8199 | 0.7737 | 0.7961 | 0.6267 |
| LogP_ECFP4_XGBoost_cls_prob | 0.8146 | 0.8881 | 0.8198 | 0.7748 | 0.7967 | 0.6274 |
| SA_ECFP4_XGBoost_cls_prob | 0.8127 | 0.8875 | 0.8186 | 0.7713 | 0.7943 | 0.6236 |
| ECFP4_XGBoost_cls_prob | 0.8134 | 0.8874 | 0.8185 | 0.7735 | 0.7953 | 0.6249 |
| XGBoost_cls_prob | 0.7982 | 0.8868 | 0.7942 | 0.7686 | 0.7812 | 0.5943 |
| SA_LogP_XGBoost_cls_prob | 0.8111 | 0.8866 | 0.8163 | 0.7704 | 0.7927 | 0.6203 |
| QED_MW_LogP_XGBoost_cls_prob | 0.8106 | 0.8863 | 0.8093 | 0.7798 | 0.7942 | 0.6193 |
| SA_XGBoost_cls_prob | 0.8080 | 0.8859 | 0.8122 | 0.7681 | 0.7896 | 0.6142 |
| QED_MW_XGBoost_cls_prob | 0.8093 | 0.8856 | 0.8070 | 0.7795 | 0.7930 | 0.6166 |
| LogP_XGBoost_cls_prob | 0.7987 | 0.8850 | 0.7954 | 0.7684 | 0.7816 | 0.5954 |
| MW_LogP_XGBoost_cls_prob | 0.8098 | 0.8847 | 0.8085 | 0.7789 | 0.7934 | 0.6177 |
| QED_XGBoost_cls_prob | 0.8030 | 0.8840 | 0.7997 | 0.7736 | 0.7864 | 0.6041 |
| QED_LogP_XGBoost_cls_prob | 0.8032 | 0.8840 | 0.8005 | 0.7728 | 0.7864 | 0.6044 |
| MW_XGBoost_cls_prob | 0.8059 | 0.8834 | 0.8021 | 0.7778 | 0.7898 | 0.6098 |
| SA_MW_ECFP4 | 0.5774 | 0.5949 | 0.5647 | 0.4305 | 0.4886 | 0.1433 |
| SA_QED_MW_LogP_ECFP4 | 0.5787 | 0.5944 | 0.5670 | 0.4292 | 0.4885 | 0.1459 |
| SA_QED_MW_ECFP4 | 0.5790 | 0.5943 | 0.5677 | 0.4273 | 0.4876 | 0.1463 |
| MW_LogP_ECFP4 | 0.5794 | 0.5926 | 0.5674 | 0.4323 | 0.4907 | 0.1473 |
| QED_MW_LogP_ECFP4 | 0.5798 | 0.5919 | 0.5689 | 0.4282 | 0.4886 | 0.1481 |
| MW_ECFP4 | 0.5788 | 0.5912 | 0.5674 | 0.4278 | 0.4878 | 0.1461 |
| QED_MW_ECFP4 | 0.5781 | 0.5903 | 0.5675 | 0.4207 | 0.4832 | 0.1444 |
| SA_MW_LogP | 0.5722 | 0.5873 | 0.5602 | 0.4069 | 0.4714 | 0.1316 |
| SA_MW | 0.5721 | 0.5869 | 0.5600 | 0.4072 | 0.4716 | 0.1314 |
| SA_QED_MW_LogP | 0.5710 | 0.5869 | 0.5591 | 0.4015 | 0.4674 | 0.1289 |
| SA_QED_MW | 0.5702 | 0.5865 | 0.5582 | 0.3992 | 0.4655 | 0.1273 |
| MW | 0.5679 | 0.5864 | 0.5545 | 0.3986 | 0.4638 | 0.1224 |
| MW_LogP | 0.5678 | 0.5863 | 0.5543 | 0.3986 | 0.4637 | 0.1222 |
| QED_MW | 0.5672 | 0.5861 | 0.5541 | 0.3934 | 0.4601 | 0.1207 |
| QED_MW_LogP | 0.5665 | 0.5861 | 0.5530 | 0.3926 | 0.4592 | 0.1191 |
| SA_MW_LogP_ECFP4 | 0.5738 | 0.5829 | 0.5622 | 0.4108 | 0.4747 | 0.1350 |
| SA_QED_LogP_ECFP4 | 0.5633 | 0.5656 | 0.5529 | 0.3583 | 0.4348 | 0.1113 |
| SA_LogP_ECFP4 | 0.5464 | 0.5601 | 0.5250 | 0.3414 | 0.4138 | 0.0745 |
| SA_QED_ECFP4 | 0.5546 | 0.5564 | 0.5389 | 0.3463 | 0.4216 | 0.0922 |
| QED_LogP_ECFP4 | 0.5535 | 0.5494 | 0.5407 | 0.3158 | 0.3987 | 0.0885 |
| SA_ECFP4 | 0.5296 | 0.5492 | 0.4973 | 0.3081 | 0.3805 | 0.0365 |
| QED_ECFP4 | 0.5488 | 0.5459 | 0.5321 | 0.3116 | 0.3930 | 0.0780 |
| QED_LogP | 0.5510 | 0.5440 | 0.5473 | 0.2443 | 0.3378 | 0.0810 |
| SA_QED_LogP | 0.5519 | 0.5426 | 0.5491 | 0.2475 | 0.3412 | 0.0833 |
| LogP_ECFP4 | 0.5130 | 0.5384 | 0.4639 | 0.2502 | 0.3251 | -0.0057 |
| LogP | 0.5333 | 0.5363 | 0.5077 | 0.1477 | 0.2289 | 0.0310 |
| ECFP4 | 0.5070 | 0.5361 | 0.4495 | 0.2293 | 0.3037 | -0.0217 |
| SA_LogP | 0.5339 | 0.5358 | 0.5098 | 0.1482 | 0.2297 | 0.0326 |
| QED | 0.5252 | 0.5336 | 0.4782 | 0.1399 | 0.2165 | 0.0075 |
| SA_QED | 0.5501 | 0.5265 | 0.5542 | 0.2059 | 0.3002 | 0.0786 |
| SA | 0.5299 | 0.5078 | 0.0400 | 0.0001 | 0.0002 | -0.0328 |

*SA, synthetic accessibility score; QED, quantitative estimates of drug-likeness; MW, molecular weight; ECFP4, recovery score of ECFP4-based anomaly detection model; XGBoost_cls_prob, classification probability for XGBoost

**Table S8. Feature importance of RF classification model and statistics for ECFP4 components**

| **ECFP4 bit*** | **Feature importance (RF)** | **Mean**  **ZINC-250k** | **SD**  **ZINC-250k** | **Mean**  **cheML.io** | **SD**  **cheML.io** | **Log2 (cheML.io/ZINC-250k)**** |
| --- | --- | --- | --- | --- | --- | --- |
| ECFP4_1171 | 0.050078 | 0.074277 | 0.156365 | 0.363201 | 0.262221 | 0.106232 |
| ECFP4_0001 | 0.030567 | 0.322344 | 0.443179 | 0.496761 | 0.467374 | 0.126153 |
| ECFP4_1057 | 0.024893 | 0.819428 | 0.886067 | 0.31773 | 0.384663 | 0.051895 |
| ECFP4_1114 | 0.024585 | 0.119832 | 0.174248 | 0.379322 | 0.324765 | 0.068454 |
| ECFP4_1060 | 0.024276 | 0.122588 | 0.18153 | 0.385456 | 0.327963 | 0.073828 |
| ECFP4_1039 | 0.022041 | 0.175394 | 0.264299 | 0.440959 | 0.380304 | 0.105193 |
| ECFP4_0283 | 0.016366 | 0.228127 | 0.327109 | 0.469157 | 0.419625 | 0.111826 |
| ECFP4_1243 | 0.015221 | 0.052759 | 0.017872 | 0.132488 | 0.223553 | -0.04862 |
| ECFP4_1911 | 0.014716 | 0.042958 | 0.008806 | 0.093425 | 0.202763 | -0.04803 |
| ECFP4_1685 | 0.012718 | 0.022652 | 0.050564 | 0.219106 | 0.148791 | 0.038849 |
| ECFP4_1989 | 0.011574 | 0.060684 | 0.031974 | 0.175932 | 0.23875 | -0.03959 |
| ECFP4_0547 | 0.010233 | 0.006291 | 0.02267 | 0.148851 | 0.079066 | 0.023294 |
| ECFP4_0397 | 0.00927 | 0.032487 | 0.055858 | 0.229648 | 0.17729 | 0.032292 |
| ECFP4_1200 | 0.008923 | 0.006905 | 0.020879 | 0.142979 | 0.08281 | 0.019884 |
| ECFP4_0794 | 0.008316 | 0.04472 | 0.017187 | 0.129967 | 0.206688 | -0.03853 |
| ECFP4_1816 | 0.008107 | 0.226242 | 0.194303 | 0.395663 | 0.418398 | -0.03807 |
| ECFP4_0926 | 0.00808 | 0.613535 | 0.649288 | 0.477193 | 0.486939 | 0.031619 |
| ECFP4_1919 | 0.007895 | 0.00447 | 0.017924 | 0.132675 | 0.066707 | 0.019195 |
| ECFP4_0225 | 0.007882 | 0.003664 | 0.016295 | 0.126608 | 0.060418 | 0.018043 |
| ECFP4_0667 | 0.007815 | 0.066591 | 0.034611 | 0.182792 | 0.249312 | -0.04392 |
| ECFP4_1274 | 0.007438 | 0.05902 | 0.086666 | 0.281345 | 0.235663 | 0.037178 |
| ECFP4_1386 | 0.006799 | 0.041764 | 0.059569 | 0.236687 | 0.200049 | 0.024449 |
| ECFP4_1946 | 0.006664 | 0.022004 | 0.040774 | 0.197765 | 0.146695 | 0.026256 |
| ECFP4_0690 | 0.006431 | 0.013294 | 0.029653 | 0.169629 | 0.114532 | 0.023105 |
| ECFP4_1923 | 0.006361 | 0.021296 | 0.041084 | 0.198485 | 0.144368 | 0.027686 |
| ECFP4_0237 | 0.006011 | 0.04884 | 0.08048 | 0.272034 | 0.215533 | 0.042878 |
| ECFP4_1145 | 0.005914 | 0.105753 | 0.060482 | 0.238378 | 0.307521 | -0.06031 |
| ECFP4_1022 | 0.005911 | 0.005715 | 0.016446 | 0.127184 | 0.075383 | 0.015312 |
| ECFP4_0782 | 0.005883 | 0.00714 | 0.020303 | 0.141035 | 0.084195 | 0.018734 |
| ECFP4_0470 | 0.005878 | 0.005263 | 0.018326 | 0.134126 | 0.072356 | 0.018626 |
| ECFP4_0261 | 0.005322 | 0.006786 | 0.018111 | 0.133355 | 0.082096 | 0.016139 |
| ECFP4_1747 | 0.005316 | 0.009946 | 0.021271 | 0.144286 | 0.099234 | 0.016087 |
| ECFP4_0229 | 0.005307 | 0.003698 | 0.013179 | 0.11404 | 0.060697 | 0.013564 |
| ECFP4_2033 | 0.005306 | 0.026346 | 0.038026 | 0.191259 | 0.160161 | 0.016326 |
| ECFP4_1088 | 0.005197 | 0.529832 | 0.600409 | 0.489814 | 0.499109 | 0.065067 |
| ECFP4_1769 | 0.005168 | 0.011964 | 0.027434 | 0.163343 | 0.108722 | 0.021888 |
| ECFP4_1017 | 0.005143 | 0.04916 | 0.067002 | 0.250025 | 0.216201 | 0.024329 |
| ECFP4_0294 | 0.005051 | 0.208329 | 0.233322 | 0.422946 | 0.406113 | 0.029537 |
| ECFP4_1366 | 0.004847 | 0.014275 | 0.023977 | 0.152978 | 0.118623 | 0.013734 |
| ECFP4_0384 | 0.004723 | 0.028973 | 0.047041 | 0.211727 | 0.16773 | 0.025113 |
| ECFP4_0650 | 0.004662 | 0.825881 | 0.860325 | 0.34665 | 0.379211 | 0.026961 |
| ECFP4_0094 | 0.004479 | 0.049624 | 0.029705 | 0.169772 | 0.217168 | -0.02764 |
| ECFP4_0184 | 0.004412 | 0.066399 | 0.047931 | 0.21362 | 0.248978 | -0.0252 |
| ECFP4_2009 | 0.004391 | 0.130384 | 0.102728 | 0.303604 | 0.336726 | -0.03574 |
| ECFP4_0807 | 0.004301 | 0.771996 | 0.803322 | 0.397486 | 0.419545 | 0.025282 |
| ECFP4_0314 | 0.004205 | 0.179471 | 0.188537 | 0.391141 | 0.383746 | 0.011047 |
| ECFP4_0316 | 0.00416 | 0.006526 | 0.017818 | 0.132288 | 0.080517 | 0.016095 |
| ECFP4_0843 | 0.004003 | 0.086176 | 0.06362 | 0.244075 | 0.280624 | -0.03028 |
| ECFP4_0378 | 0.003961 | 0.467984 | 0.478714 | 0.499547 | 0.498974 | 0.010507 |
| ECFP4_1162 | 0.003896 | 0.070135 | 0.08845 | 0.283949 | 0.255375 | 0.024482 |
| ECFP4_1152 | 0.003855 | 0.56571 | 0.601352 | 0.48962 | 0.495663 | 0.032473 |
| ECFP4_1773 | 0.003805 | 0.021334 | 0.012835 | 0.112563 | 0.144495 | -0.01206 |
| ECFP4_0786 | 0.003739 | 0.00601 | 0.01638 | 0.126931 | 0.077288 | 0.014796 |
| ECFP4_0446 | 0.003736 | 0.048921 | 0.031013 | 0.173353 | 0.215702 | -0.02484 |
| ECFP4_1019 | 0.003702 | 0.473849 | 0.483713 | 0.499735 | 0.499316 | 0.009624 |
| ECFP4_0875 | 0.003583 | 0.443818 | 0.398535 | 0.489597 | 0.496834 | -0.04597 |

*Highest 56 bits are listed, of which cumulative feature importance is over 0.5

** Log_2_ fold change value has been calculated using the mean of cheML.io over that of ZINC-250k, with a pseudocount of 1.0.

**Table S9. Co-occurrence of bit pairs for the 12 bits with the highest feature importance for ZINC-250k and cheML.io datasets**

| **ECFP4 bit** | | **All (*n* = 836,787)** | | | **ZINC-250k (*n* = 234,461)** | | | **cheML.io (*n* = 602,326)** | | |
| --- | --- | --- | --- | --- | --- | --- | --- | --- | --- | --- |
| **Bit a** | **Bit b** | **X*** | **F**** | **C/E***** | **X*** | **F**** | **C/E***** | **X*** | **F**** | **C/E***** |
| 1243 | 1911 | inf | inf | 4.4271 | inf | inf | 3.0787 | 54.7399 | 38.9547 | 2.5951 |
| 1060 | 1685 | inf | inf | 2.8251 | inf | 271.1203 | 2.4683 | inf | inf | 2.7253 |
| 0001 | 0283 | inf | inf | 2.4158 | inf | inf | 3.0668 | inf | inf | 2.2314 |
| 1060 | 0547 | inf | inf | 2.0764 | 38.5735 | 31.9355 | 1.9135 | inf | inf | 1.9518 |
| 1911 | 1989 | 116.0550 | 93.5114 | 1.8998 | 54.3188 | 47.1129 | 1.6001 | 0.9948 | 1.0022 | 1.1262 |
| 1114 | 1060 | inf | inf | 1.8641 | inf | inf | 2.1006 | inf | inf | 1.7683 |
| 1171 | 1114 | inf | inf | 1.8267 | 24.0048 | 25.3969 | 0.7969 | inf | inf | 1.8738 |
| 1060 | 1989 | inf | inf | 1.7565 | inf | inf | 2.1661 | inf | inf | 1.6956 |
| 1243 | 1989 | 76.7352 | 66.1107 | 1.5921 | 34.0348 | 30.7212 | 1.4241 | 3.0660 | 3.0004 | 1.1766 |
| 1171 | 1060 | inf | inf | 1.4018 | 0.2405 | 0.2422 | 0.9888 | inf | inf | 1.3864 |
| 1114 | 0547 | 91.4895 | 84.0088 | 1.3776 | 3.6308 | 3.4724 | 1.2617 | 58.5003 | 54.7520 | 1.2991 |
| 0001 | 1989 | 204.1308 | 201.1681 | 1.1965 | 270.5031 | 256.8633 | 1.4146 | 90.8153 | 90.2240 | 1.1613 |
| 1114 | 1243 | 36.5800 | 35.0292 | 1.1904 | 85.3307 | 76.7645 | 1.4666 | 16.4547 | 15.8447 | 1.1755 |
| 0283 | 1989 | 118.0070 | 114.4449 | 1.1898 | 142.8601 | 133.1931 | 1.3815 | 70.5719 | 68.8013 | 1.1816 |
| 0283 | 0547 | 43.8570 | 42.6517 | 1.1727 | 11.8928 | 11.2807 | 1.3403 | 12.4330 | 12.3028 | 1.0883 |
| 1057 | 0283 | inf | inf | 1.1505 | inf | inf | 1.2169 | inf | inf | 1.1265 |
| 1171 | 1685 | 13.2815 | 13.0013 | 1.0994 | 0.0003 | 0.0093 | 1.0013 | 0.7888 | 0.7922 | 1.0182 |
| 0001 | 1057 | inf | inf | 1.0911 | inf | inf | 1.1403 | inf | inf | 1.0720 |
| 1114 | 1039 | 38.2434 | 37.8761 | 1.0585 | 0.4113 | 0.4150 | 1.0106 | 13.7545 | 13.6819 | 1.0359 |
| 1171 | 1039 | 16.3098 | 16.1931 | 1.0417 | 4.0788 | 4.1367 | 0.9376 | 0.2502 | 0.2520 | 1.0029 |
| 0001 | 0547 | 4.0088 | 4.0101 | 1.0378 | 1.1347 | 1.1588 | 1.0684 | 1.6709 | 1.6788 | 0.9781 |
| 1171 | 0547 | 0.8850 | 0.8914 | 1.0313 | 7.8794 | 9.4306 | 0.4746 | 1.9286 | 1.9532 | 0.9503 |
| 1039 | 1989 | 2.8674 | 2.8655 | 1.0306 | 5.2218 | 5.1537 | 1.0799 | 14.5969 | 14.3809 | 1.0937 |
| 1057 | 1060 | 54.7520 | 56.0182 | 1.0151 | 6.6180 | 6.6968 | 1.0134 | 17.5214 | 17.7905 | 1.0086 |
| 1171 | 0001 | 3.3726 | 3.3726 | 1.0118 | 27.0414 | 27.5884 | 0.8845 | 3.2782 | 3.2848 | 0.9884 |
| 1057 | 0547 | 1.8901 | 1.9107 | 1.0079 | 0.3358 | 0.3418 | 1.0094 | 1.9052 | 1.8974 | 0.9924 |
| 1057 | 1989 | 3.1013 | 3.1261 | 1.0070 | 20.7932 | 21.6635 | 1.0364 | 1.7478 | 1.7685 | 1.0061 |
| 1039 | 0547 | 0.1072 | 0.1077 | 0.9959 | 7.1068 | 7.6990 | 0.6958 | 3.4353 | 3.4685 | 0.9496 |
| 1685 | 0547 | 0.0474 | 0.0442 | 0.9943 | 1.0869 | 1.1073 | 0.6884 | 2.2428 | 2.2990 | 0.8980 |
| 1039 | 1685 | 0.3922 | 0.3945 | 0.9923 | 11.5031 | 12.1549 | 0.7944 | 4.0301 | 4.0545 | 0.9635 |
| 1057 | 1039 | 34.4989 | 34.1403 | 0.9906 | 23.5346 | 23.1494 | 0.9786 | 68.9788 | 67.7620 | 0.9864 |
| 1057 | 1911 | 9.6655 | 9.3990 | 0.9801 | 1.5597 | 1.5682 | 1.0101 | 0.8831 | 0.8905 | 1.0075 |
| 0001 | 1039 | 34.4597 | 34.5302 | 0.9710 | 33.4597 | 33.8356 | 0.9208 | 75.3904 | 75.5544 | 0.9555 |
| 1057 | 1114 | 200.0106 | 192.0482 | 0.9703 | 106.4413 | 101.1029 | 0.9422 | 183.9355 | 175.5467 | 0.9708 |
| 1114 | 1911 | 1.0081 | 1.0121 | 0.9694 | 4.9172 | 4.8239 | 1.1160 | 3.4535 | 3.4078 | 1.1069 |
| 1057 | 1685 | 64.4168 | 61.0381 | 0.9655 | 52.4698 | 47.8697 | 0.9021 | 69.5528 | 65.0540 | 0.9645 |
| 1171 | 0283 | 21.7773 | 21.9281 | 0.9584 | 17.2503 | 17.7055 | 0.8840 | 67.0545 | 67.7986 | 0.9253 |
| 1039 | 0283 | 45.6946 | 45.9508 | 0.9573 | 27.9066 | 28.3768 | 0.9085 | 86.8041 | 87.4112 | 0.9388 |
| 1114 | 1685 | 4.5258 | 4.5834 | 0.9502 | 12.9747 | 14.0372 | 0.7259 | 7.7932 | 7.9172 | 0.9312 |
| 1171 | 1057 | inf | inf | 0.9481 | 192.9245 | 176.0799 | 0.8984 | inf | inf | 0.9461 |
| 1060 | 1039 | 36.4815 | 36.8665 | 0.9441 | 68.5751 | 72.2168 | 0.7891 | 35.9172 | 36.2660 | 0.9423 |
| 0283 | 1685 | 12.2612 | 12.3872 | 0.9429 | 19.0400 | 20.1002 | 0.7725 | 23.0448 | 23.3665 | 0.9195 |
| 1057 | 1243 | 122.8633 | 112.0223 | 0.9400 | 22.5482 | 21.7212 | 0.9591 | 33.0899 | 30.5952 | 0.9584 |
| 0001 | 1685 | 23.7852 | 23.9586 | 0.9365 | 51.9747 | 55.2358 | 0.6992 | 30.6289 | 30.8041 | 0.9271 |
| 1114 | 0283 | 117.1662 | 118.8601 | 0.9112 | 134.9586 | 143.0590 | 0.7447 | 91.2175 | 92.2741 | 0.9182 |
| 0001 | 1114 | 248.0391 | 250.4248 | 0.8983 | 247.0872 | 259.5884 | 0.7271 | 178.8633 | 179.8894 | 0.9101 |
| 1060 | 1911 | 29.3665 | 31.0696 | 0.7950 | 4.1079 | 4.2118 | 0.8966 | 1.2137 | 1.2197 | 0.9451 |
| 1060 | 0283 | inf | inf | 0.7928 | 192.9586 | 207.1035 | 0.6982 | inf | inf | 0.7883 |
| 0001 | 1060 | inf | inf | 0.7567 | 253.0101 | 265.7235 | 0.7274 | inf | inf | 0.7436 |
| 1039 | 1243 | 103.5331 | 110.1831 | 0.7493 | 9.7167 | 9.9914 | 0.8790 | 39.4622 | 41.4449 | 0.7884 |
| 1243 | 0547 | 7.5302 | 8.1463 | 0.7339 | 0.7636 | 0.7947 | 1.1565 | 1.0818 | 1.1096 | 0.8892 |
| 1685 | 1989 | 31.6778 | 34.9788 | 0.6993 | 27.5800 | 34.5850 | 0.4096 | 3.3107 | 3.4045 | 0.8924 |
| 1989 | 0547 | 13.8697 | 15.3116 | 0.6953 | 6.0721 | 7.1605 | 0.4916 | 2.4397 | 2.5029 | 0.8635 |
| 0283 | 1243 | 223.2418 | 239.6536 | 0.6831 | 52.0376 | 55.2480 | 0.7537 | 79.8125 | 84.0057 | 0.7395 |
| 0283 | 1911 | 164.9872 | 178.0942 | 0.6645 | 53.3969 | 57.2441 | 0.7220 | 22.0894 | 22.9281 | 0.8069 |
| 1171 | 1243 | 97.7399 | 109.0610 | 0.6518 | 9.8729 | 10.4698 | 0.8010 | 27.0287 | 28.9666 | 0.7575 |
| 0001 | 1243 | inf | inf | 0.6449 | 137.8794 | 147.0953 | 0.6819 | 160.5670 | 165.7258 | 0.7102 |
| 0001 | 1911 | 304.5817 | inf | 0.6414 | 96.8894 | 102.8069 | 0.7035 | 61.0000 | 62.6216 | 0.7458 |
| 1039 | 1911 | 142.8182 | 157.4413 | 0.6365 | 30.3768 | 32.4776 | 0.7546 | 33.7212 | 36.0757 | 0.7205 |
| 1060 | 1243 | 138.0506 | 154.2140 | 0.6342 | 51.6925 | 57.6716 | 0.6430 | 36.8356 | 39.6180 | 0.7400 |
| 1171 | 1911 | 75.5751 | 85.3080 | 0.6232 | 2.1138 | 2.1590 | 0.9076 | 17.5884 | 19.0227 | 0.7222 |
| 1171 | 1989 | 189.3478 | 215.3595 | 0.5985 | 54.2299 | 63.1707 | 0.5517 | 76.2549 | 83.4461 | 0.6940 |
| 1911 | 1685 | 27.2941 | 31.6737 | 0.5843 | 4.6126 | 5.0074 | 0.7276 | 2.7542 | 2.8811 | 0.8129 |
| 1114 | 1989 | 276.1175 | inf | 0.5621 | 161.6402 | 200.1785 | 0.4018 | 90.2007 | 98.6904 | 0.6878 |
| 1243 | 1685 | 47.0119 | 55.2132 | 0.5542 | 5.5850 | 6.0458 | 0.7280 | 18.1337 | 20.5086 | 0.6320 |
| 1911 | 0547 | 16.8069 | 20.2441 | 0.4964 | 3.6968 | 4.2233 | 0.5366 | 0.8397 | 0.8598 | 0.8649 |

* Negative log 10 of *p*-value for *X^2^* test

** Negative log 10 of *p*-value for Fisher’s exact test

*** Ratio for observed co-occurrence over expected

## **References**

[1] Irwin JJ, Tang KG, Young J, Dandarchuluun C, Wong BR *et al.* (2020) ZINC20—A Free Ultralarge-Scale Chemical Database for Ligand Discovery. J Chem Inf Model 60:6065-6073.

[2] Zhumagambetov R, Kazbek D, Shakipov M, Maksut D, Peshkov VA *et al.* (2020) cheML.io: an online database of ML-generated molecules. RSC Adv 10:45189-45198.

[3] Ertl P, Schuffenhauer A (2009) Estimation of synthetic accessibility score of drug-like molecules based on molecular complexity and fragment contributions. J Cheminform 1:8.

[4] Rogers D, Hahn M (2010) Extended-Connectivity Fingerprints. J Chem Inf Model 50:742-754.

[5] Polykovskiy D, Zhebrak A, Sanchez-Lengeling B, Golovanov S, Tatanov O *et al.* (2020) Molecular Sets (MOSES): A Benchmarking Platform for Molecular Generation Models. Front Pharmacol 11:565644.

[6] Makhzani A, Shlens J, Jaitly N, Goodfellow I, Frey B (2015) Adversarial autoencoders. arXiv preprint arXiv:1511.05644.

[7] Kingma DP, Welling M (2013) Auto-encoding variational bayes. arXiv preprint arXiv:1312.6114.

[8] Segler MHS, Kogej T, Tyrchan C, Waller MP (2018) Generating Focused Molecule Libraries for Drug Discovery with Recurrent Neural Networks. ACS Cent Sci 4:120-131.

[9] Jin W, Barzilay R, Jaakkola T (2018) Junction tree variational autoencoder for molecular graph generation. In: International conference on machine learning. PMLR; 2018. pp. 2323-2332.

[10] Prykhodko O, Johansson SV, Kotsias P-C, Arús-Pous J, Bjerrum EJ *et al.* (2019) A de novo molecular generation method using latent vector based generative adversarial network. J Cheminform 11:1-13.
